# Interpretation of vaccine associated neurological adverse events: a methodological and historical review

**DOI:** 10.1007/s00415-021-10747-8

**Published:** 2021-08-16

**Authors:** Marija Cauchi, Harriet Ball, Yoav Ben-Shlomo, Neil Robertson

**Affiliations:** 1 Division of Psychological Medicine and Clinical Neuroscience, Department of Neurology, University Hospital of Wales, Cardiff University, Heath Park, Cardiff, CF14 4XN UK; 2 Population Health Sciences, Bristol Medical School, Bristol, BS8 2PS UK

**Keywords:** Vaccination, COVID-19, Thrombosis with thrombocytopenia syndrome, Vaccine-induced thrombocytosis with thrombocytopenia

## Abstract

As a result of significant recent scientific investment, the range of vaccines available for COVID-19 prevention continues to expand and uptake is increasing globally. Although initial trial safety data have been generally reassuring, a number of adverse events, including vaccine induced thrombosis and thrombocytopenia (VITT), have come to light which have the potential to undermine the success of the vaccination program. However, it can be difficult to interpret available data and put these into context and to communicate this effectively. In this review, we discuss contemporary methodologies employed to investigate possible associations between vaccination and adverse neurological outcomes and why determining causality can be challenging. We demonstrate these issues by discussing relevant historical exemplars and explore the relevance for the current pandemic and vaccination program. We also discuss challenges in understanding and communicating such risks to clinicians and the general population within the context of the ‘infodemic’ facilitated by the Internet and other media.

## Introduction

As a result of a rapid and focussed international scientific response to the COVID-19 pandemic, an impressive array of vaccines against SARS-CoV-2 is now available and the largest vaccination program in history is now well underway. However, successful global implementation will be critically dependent on the public health response, with uptake of at least 70% required to achieve “herd-immunity”. However, it can be difficult to interpret large-scale safety data, and vaccines have been historically linked to a variety of notable adverse neurological events. Many of these still dominate the popular imagination, demonstrating how difficult it can be to change public opinion on safety and avoid vaccine hesitancy after seeds of doubt have been sown.

When contemplating vaccination at such an immense global scale, it is particularly important to be vigilant for any iatrogenic harm, even when occurring at very low frequency. The precautionary principle warns against adopting new technology before sufficient evidence has accumulated about its effects. However, the risk of adopting a vaccine needs to be weighed against the population prevalence of disease, transmissibility, and the mortality and morbidity risk of the infection itself. Safety concerns should shift priority towards alternatives with similar efficacy and more robust safety data where possible but achieving an appropriate balance will depend on complex and dynamic contextual factors.

## COVID-19 vaccination: neurological adverse events

There are currently 100 vaccines in clinical development and more at a pre-clinical stage. Data available from phase III randomised clinical trials of COVID-19 vaccinations have been largely reassuring, although real-world experience has identified important issues for selected vaccines. The phase III Oxford-AstraZeneca (AZ) (Vaxzevria) vaccine trial reported a single case of transverse myelitis thought to have been related to the vaccine [1]. Two additional cases were thought unrelated; one occurred in the active control group after 68 days and another had evidence of pre-existing multiple sclerosis (MS). Whether the vaccine could have acted as a precipitant for this presenting event is difficult to determine and may warrant further investigation.

Phase III trials of vaccines using mRNA technology from Moderna and Pfizer/BioNTech (Comirnaty) reported no serious adverse neurological events, apart from a slight excess (< 0.1%) of Bell’s palsy [2–4], a condition also noted in subsequent case series [5, 6]. The Janssen COVID-19 vaccine demonstrated a similar safety profile, with three cases of Bell’s palsy, 1 Guillain-Barre syndrome (GBS), and 1 episode of brachial radiculitis occurring within 28 days of vaccination (versus none in placebo group). Venous thromboembolic events were also more common in the vaccine group (*n* = 11/21,895), one of which was a cerebral venous embolic event accompanied by thrombocytopenia, and four cases of seizures were also reported (versus 1 in the placebo group) [7].

However, whilst pre-licensure randomised clinical trials allow assessment of a vaccine’s efficacy and safety profile, it is important to note that the sample sizes of conventional vaccine trials and limited duration of follow-up are unlikely to reliably detect rare events. Monitoring of real-world vaccine experience via passive and active surveillance systems facilitates early detection, investigation, and analysis of adverse events following immunization [8] and are a key tool in developing an understanding of longer term and low frequency events as well as contextualising risks.

An example of this that could not have been detected in the phase 3 trials were thrombotic cases associated with thrombocytopenia and antibodies to PF4 reported following the AstraZeneca (AZ) and Jannsen vaccinations [9–11]. As of 9th June 2021, 390 cases of vaccine induced thrombosis and thrombocytopenia (VITT) have occurred within 28 days following vaccination with the COVID-19 Vaccine AstraZeneca in the United Kingdom where there has been a high early uptake. 67 were fatal after an estimated 24.6 million first doses; (2.7 deaths/million first-dose vaccinations). Cerebral venous sinus thrombosis (CVST) was reported in 140 of these cases and 250 had other major thromboembolic events with concurrent thrombocytopenia [12]. Current data seem to indicate a higher risk in younger populations; with possibly a higher incidence in females. In the UK, a small number of possible cases have also been reported to occur within 28 days of Pfizer vaccination (reported under peer review).

## Determining if Vaccine Side Effects are Causal

Establishing a causal association between vaccination and an adverse outcome is no different than any other causal association, except that the exposure (vaccination) is well-classified and “temporal” relationship between exposure and outcome well defined; hence, there is no concern about reverse causation. The Bradford Hill criteria are frequently used to guide decisions [13], but other than temporality are not essential, and may even be misleading if studies finding similar effects are “consistently” biased. Apart from chance associations, modern causal thinking [14] considers three reasons for non-causal associations that may mislead; (i) confounding, (ii) collider bias (including selection of individuals into the analysis^1^), and (iii) measurement bias. These problems are more pertinent for observational studies, but may also apply to poorly designed or executed randomised-controlled trials (RCT).

## Experimental studies: randomised-controlled trials (RCTs)

In an RCT, the randomisation of participants to either a treatment or control arm should avoid confounding, since all factors other than the exposure should be balanced across groups. This is true provided that the sample size is sufficiently large (as in phase III RCTs) and there is adequate randomisation which ensures concealment of allocation. Adequate blinding (where neither participants, clinicians, or outcome assessors are aware of the treatment) also avoids differential measurement error such as recall or detection bias. However, participants or clinicians may be able to determine treatment despite efforts to blind (e.g., cannabinoids for spasticity in MS) [15]. The use of an active control (such as meningitis vaccine, rather than an inert placebo, as used with the AZ COVID vaccine) helps maintain blinding more effectively. To avoid selection bias, an intention to treat analysis is undertaken, but excellent follow-up is still required; otherwise, imputation methods will need to be employed. In addition, whilst most RCTs are powered to detect common side effects such as headache, they will never have sufficient power to detect very rare events such as GBS or VITT, whose detection is therefore dependent on alternative methodologies (see Table 1).

**Table 1:** Incidence rates influence the feasibility of different study designs

Condition	Background incidence per 1000 person years	Minimum sample size needed in each arm of a hypothetical RCT^a^	Appropriate study design, considering the incidence
Low back pain	15–360 [93]	150	RCT
Single unprovoked seizure	0.23–0.61 [94]	150,000	Data linkage studies, e.g., CPRD; or self-controlled case series
Cerebral venous sinus thrombosis	0.01–0.02 [11]	5,000,000	National surveillance, e.g., registry of all cases in all of Denmark & Norway

*CPRD* Clinical Practice Research Datalink (UK)

^a^Sample size needed to detect a 50% increase. Assuming alpha 0.05, power 80%, follow-up for 1 year

### Observational studies

Observational studies involve no investigator-led manipulation of exposure but attempt to uncover effects of an exposure by comparing outcomes for people who have or who have not been exposed. They are more prone to bias than experimental studies and can lead to clinical and public concern with adverse health consequences. A highly publicised editorial in the British Medical Journal [16] which suggested myopathy was common with statin treatment (seen in observational but not in the RCTs), led to patients stopping statin therapy, with an estimated overall increase in future cardiovascular events of at least 2173 over the following 10 years [17].

#### Cohort and case–control designs

Cohort studies involve a group of people unselected for the disorder in question, whilst case–control studies purposively sample subjects with and without the disorder and are the most efficient design for rare outcomes (e.g., Creutzfeldt–Jakob disease) [18] especially if new data have to be collected. Recall bias is not relevant for vaccine studies as the exposure is usually available from medical records. Large cohort studies can be created using routine datasets with record linkage. For example, a study found no association between MMR vaccination and autism by linking vaccination status with the Psychiatric Central Register for all children in Denmark [19]. The usual challenge with this approach is to adjust for confounders that may differ between those getting vaccinated (or vaccinated early) and those not vaccinated (or vaccinated late) that themselves maybe associated with the outcome producing a non-causal association. There are several statistical approaches (e.g., propensity matching) to help take this into account, but all rely on adequate measurement of potential confounders.

#### Alternative study designs

**Fig. 1:**
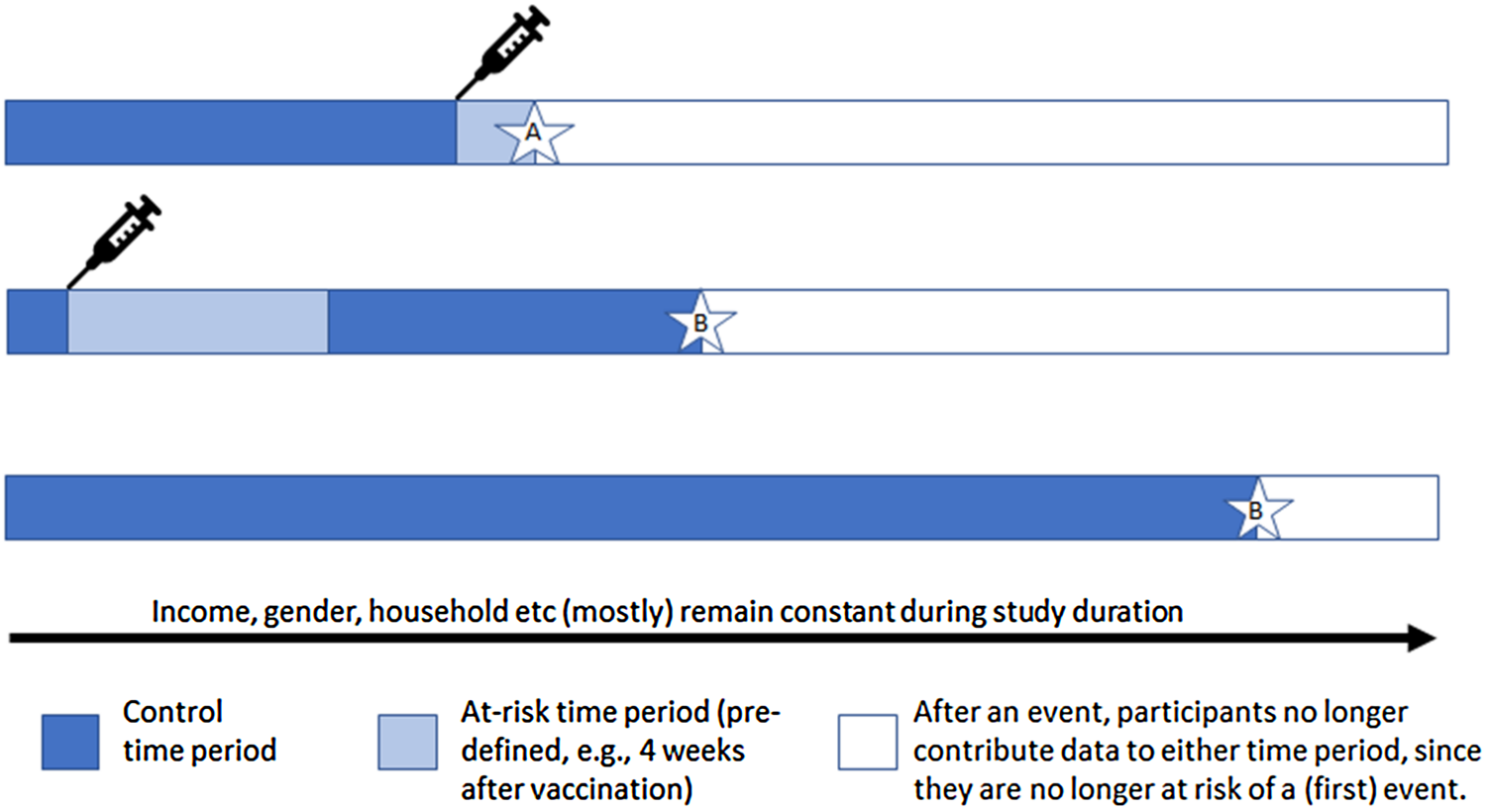
Self-controlled case series: example timelines of three participants. Averaged across all people in the study, the rate of events **A** occurring in the at-risk time period is compared to the rate of events **B** in the control time period. Many factors which in other designs could be confounders are assumed to be constant within each person across their duration of participation. Only those who have experienced an event are included as cases

Case-crossover and self-controlled case series (SCCS) designs [20] are rarely used methods that can be particularly useful for short-term adverse effects, such as investigating vaccine side effects. They are equivalent to case–control and cohort studies, but subjects who are cases or who are exposed, also act as their own controls or unexposed individuals (see Fig. 1). This is especially helpful where data on confounders are absent or poorly measured, but it assumes that confounders are fixed over a short time. An SCCS study of myocardial infarction observed a three-to-fourfold increased risk ratio in the first week post-acute infection (but not post-vaccination), an effect which disappeared with longer risk periods [21].

#### Ecological studies, natural experiments, and time series analyses

Ecological studies (which compare data at the group not the individual level) are usually regarded as low on the hierarchy of evidence as they are prone to the ecological fallacy (confounding at a group level). However, in relation to vaccine exposure, they may be conceptualised as a natural experiment. If the choice of vaccine or speed of delivery is related to political factors and not the risk of the outcome in the population, then individual confounding should not be present, similar to an RCT. For example, rare cases of VITT may have become evident in some countries before others, due to the earlier use of the AZ vaccine in younger age groups in certain countries. Countries like Israel which have only used the Pfizer vaccine can act as a control population for other countries that have also used AZ such as the UK, although there may be racial/ethnic differences in risk of outcomes across countries. Within-country analyses can also be performed using interrupted time series analyses (i.e., examining rates of a disorder, at the population level, before and after an index date) with vaccine licensing as the index date. This is more problematic when several vaccines are used concurrently.

#### Case series and surveillance systems (EudraVigilance; Yellow Card)

**Fig. 2:**
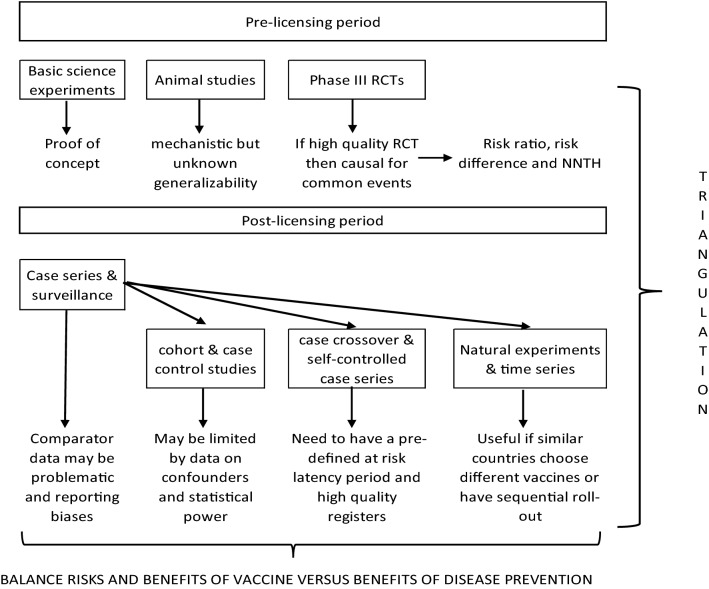
Hypothetical illustration of how potential vaccine-related complications could be investigated. NNTH = number needed to treat for harm

Individual case reports have no capacity to assess whether a vaccination and adverse event are causally linked, except by appealing to the temporal interval and biological plausibility (e.g., an episode of anaphylaxis within 15 min of vaccination). Case series ideally describe all consecutive cases of a disorder presenting to a clinical service, with the aim of discovering shared predictors that could be causes. Near-real-time pharmacovigilance databases (such as Yellow Card in the UK, and EudraVigilance) are set up with the aim of rapidly identifying any possible safety concerns. These are essentially very large-scale case series, which enable post-marketing surveillance, and are especially useful for rare events. Following the identification of a possible signal, epidemiological studies should then be performed to test and quantify any potential cause–effect relationships in a more rigorous fashion (see Fig. 2 below).

### Accounting for functional neurological disorder (FND) reactions following vaccination

Non-placebo-controlled studies need to consider the possibility of “nocebo” events (adverse effects experienced and reported in the context of negative expectations around an intervention). Functional Neurological Disorder (FND) reactions to vaccination can be conceptualised as a complex reaction to a nocebo effect. Heightened stress levels during pandemics, feelings of uncertainty around the vaccine, and transient physical discomfort likely combine to make such reactions more common. These may be amplified by intense media interest focussed on neurological symptoms (initially reported as unexplained) [22, 23]. This means that such cases are considerably more likely to show up in post-marketing surveillance than in the initial trials [23, 24]. Unfortunately, systematic incidence data are lacking; at best a case (listed as FND or conversion disorder) will be mentioned in passing in studies set up to look for other conditions [25–27]. Such events are more likely to occur in settings where many (especially young) patients are vaccinated at once [28]. Clusters of cases have led to delayed rollout of vaccination programs [29]. One study from Rochester, New York (1978) collated systematic reporting of post-vaccination concerns reported to a local health clinic. This study began the morning after the swine flu vaccination was cancelled due to GBS fears, at which time there was much media speculation detailing neurological symptoms and their potential connection to the vaccine [30]. The rate of reactions found was far higher than the number of GBS cases separately documented in the wider area, though none of the conversion reactions had come to the attention of the local neurologist.

FND symptoms appear to be common post-vaccination and may have a high healthcare burden. They can be challenging to diagnose because of the wide variety of potential symptoms, often presenting to non-specialists and are often under-reported. Greater clarity on diagnosis and systematic reporting should allow quantification, with benefits for patient education as well as wider scientific and social understanding of functional disorders.

## Lessons learnt from history

The long history of vaccination provides some important lessons in determining (non) causal links between vaccination and illnesses. This section provides selected historical exemplars, which remain of value when considering emerging data from contemporary vaccination programs.

### Contextualising risk: influenza vaccination and Guillain-Barre syndrome

In 1976, an unexpected increase in GBS cases was linked to a mass vaccination program against swine flu, resulting in its suspension in the United States [31]. Later, during the 2009 H1N1 influenza pandemic, a high degree of public interest in the safety of the vaccine (also of swine origin) saw the adoption of safety surveillance systems [32]. Several studies have since addressed the possible association between seasonal or pandemic influenza vaccinations and GBS resulting in varying risk estimates; most of which are consistent with chance variation. Contemporary meta-analyses have reported pooled risk estimates of 1.15–1.22 (95% CI 0.97–1.35; 1.01–1.48) [33, 34]. More recently, an SCCS analysed all GBS cases occurring in influenza seasons 2010–2014 [35]. Incidence of GBS in the exposure period (day 1–42 following vaccination) was compared to that in the control period and no increased risk of GBS was identified (Risk Ratio_crude_ 1.02; 95% CI 0.83–1.25; *p* = 0.85). Conversely a large systematic review and meta-analysis in 2020 found that previous influenza-like illness was significantly associated with GBS, with an effect size of 9.6 (95% CI 4.0–23.0) [33], highlighting the much larger risk in unvaccinated populations.

It is thought that there may be an increased risk for developing GBS from pandemic influenza vaccinations such as H1N1 (RR = 1.84; 95% CI 1.36–2.50) compared to seasonal vaccination (RR = 1.22; 95% CI 1.01–1.48) [34]. This finding is in line with the other studies showing a slight increased risk of GBS [36–42]. However, other studies have failed to confirm this association [43–45].

Overall, there appears to be a consensus for a small association between influenza vaccines and GBS, but the risk is consistently very low (~ 1–3 additional GBS cases per million vaccinations) [46] and needs to be carefully weighed against evidence supporting benefits of vaccination in preventing influenza-related complications, including an increased risk of GBS [43, 47].

Key points

- Large-scale prospective surveillance registers can offer key data in comparative estimations of risk.
- There is rarely a zero-risk option; the risk of complications from preventable disease may be higher than risk of complications from vaccination itself, particularly in periods of high disease transmission.

### Consequences of a false-positive signal: hepatitis B vaccination and multiple sclerosis

In the early 1990s, French authorities undertook an immunization programme against hepatitis B (HB) targeting at-risk adults, infants, and pre-adolescents. Case reports then began to emerge raising concerns that HB vaccination was linked to autoimmune diseases, including Multiple Sclerosis (MS). Subsequently, case–control studies [48, 49] identified a slight increase in the risk of MS/demyelination in the months following HB vaccination, leading to suspension of the school-based adolescent vaccination programme in 1998. 624 demyelination cases were reported to the French Medicines Agency (FMA) between 1981 and 2000 possibly linked to HB vaccination of which 422 were a first episode of MS [50]. However, it is important to note that although the vaccination program was targeted at new-borns, children, and at-risk adults, a disproportionately higher number of adolescents and young adults were vaccinated—at an age where demyelination risk is highest [50]. Expert consensus panels organised by the French authorities failed to confirm an increased risk [51–54], but the damage in public confidence resulted in a legacy of distrust and low vaccination rates [55, 56], which even now may be reflected in the current national COVID-19 vaccination program [57].

Although a small number of case–control studies have since reported an increase in risk of a first demyelinating event in the first few months following vaccination [58–60], the majority of case–control and observational studies have failed to demonstrate a significantly increased risk [60–69]. A French MS Society task force set up in 2017 eventually concluded that there was no evidence for an association [70] and recent meta-analyses have supported this position [71]. Only one study has addressed the risk of MS relapse with HBV, and also failed to identify an association [66].

Key points

- Vaccination programs often target at-risk groups which may have a higher background incidence rate of an event than the rest of the population.
- Initial unresolved reports can have profound and lasting consequences for public confidence. Data analysis should focus on the highest-quality studies and the best amalgamation of evidence.

### Expect the unexpected: Pandremix© vaccination and narcolepsy

In April 2009, the H1N1 A/California pandemic influenza strain spread globally. Rapid development of influenza vaccines followed. Pandremix, an adjuvanted vaccine produced by GlaxoSmithKline (GSK), was administered with over 31 million doses primarily in Europe [72]. By August 2010, a possible association between narcolepsy and Pandremix had been reported in Sweden and Finland [73, 74], resulting in epidemiological studies being set up across Europe.

Following analysis of data from a retrospective cohort study in 2012 [75], the Finnish National Institute for Health and Welfare estimated the relative risk for narcolepsy after Pandremix in children aged 4–19 was large at 12.7 (95% CI 6.1–30.8). International retrospective cohort studies performed on newly diagnosed narcolepsy patients [75–81] and a recent meta-analysis have estimated an increase in relative risk of 5–14-fold in vaccinated children and adolescents, and 3–8-fold in adults within the first year of vaccination with Pandremix [82]. Studies conducted in South Korea and the United States using alternative H1N1 vaccines did not report an increased risk [83, 84]. Retrospective cohort and case-control studies have also identified an increased incidence of narcolepsy following influenza [85, 86], although others have shown that this was not the main factor contributing to narcolepsy [87].

The Pandremix and narcolepsy association illustrates an appropriately investigated observed signal with confirmation across multiple studies, despite a potential for ascertainment bias [88], and several countries now offer compensation for Pandremix-associated narcolepsy.[89].

Key points

- International systems should be in place to rapidly investigate possible rare adverse events.
- It may be problematic to distinguish adverse events occurring during periods of high background infection rates from those secondary to vaccination.

## How to make sense of emerging data

Many contextual factors need to be considered when evaluating concerns around possible vaccine side effects. The background rate of a particular outcome will affect the power to detect an observed increase from the background rate (Table 1). This is problematic for rare events, even in a generously sized RCT, necessitating different approaches.

Following licensing, post-marketing surveillance is crucial to detect signals of potential concern. One drawback of these surveillance systems is the need for an appropriate comparator group. Considerable difficulties arose when evaluating early signals of VITT, since prevalence estimates of CVST are scarce, let alone the highly unusual combination of abnormalities in VITT. Finding a rate higher than the background can be misleading, for example through ascertainment or reporting bias (e.g., a patient or clinician being more mindful of the potential for novel side effects in the context of scientific and media interest in the new vaccine). Therefore, where concern remains, this needs investigation via a study design that has a larger sample size than would be possible in an RCT, an adequate comparator population or using a self-controlled design (see Fig. 2).

In a pandemic situation, it is also necessary to consider whether a newly prevalent symptom could be the outcome of the infection rather than vaccination [90]. This might alter the expected baseline incidence compared to studies conducted in pre-pandemic times.

## Fighting misinformation in the age of social media

**Fig. 3:**
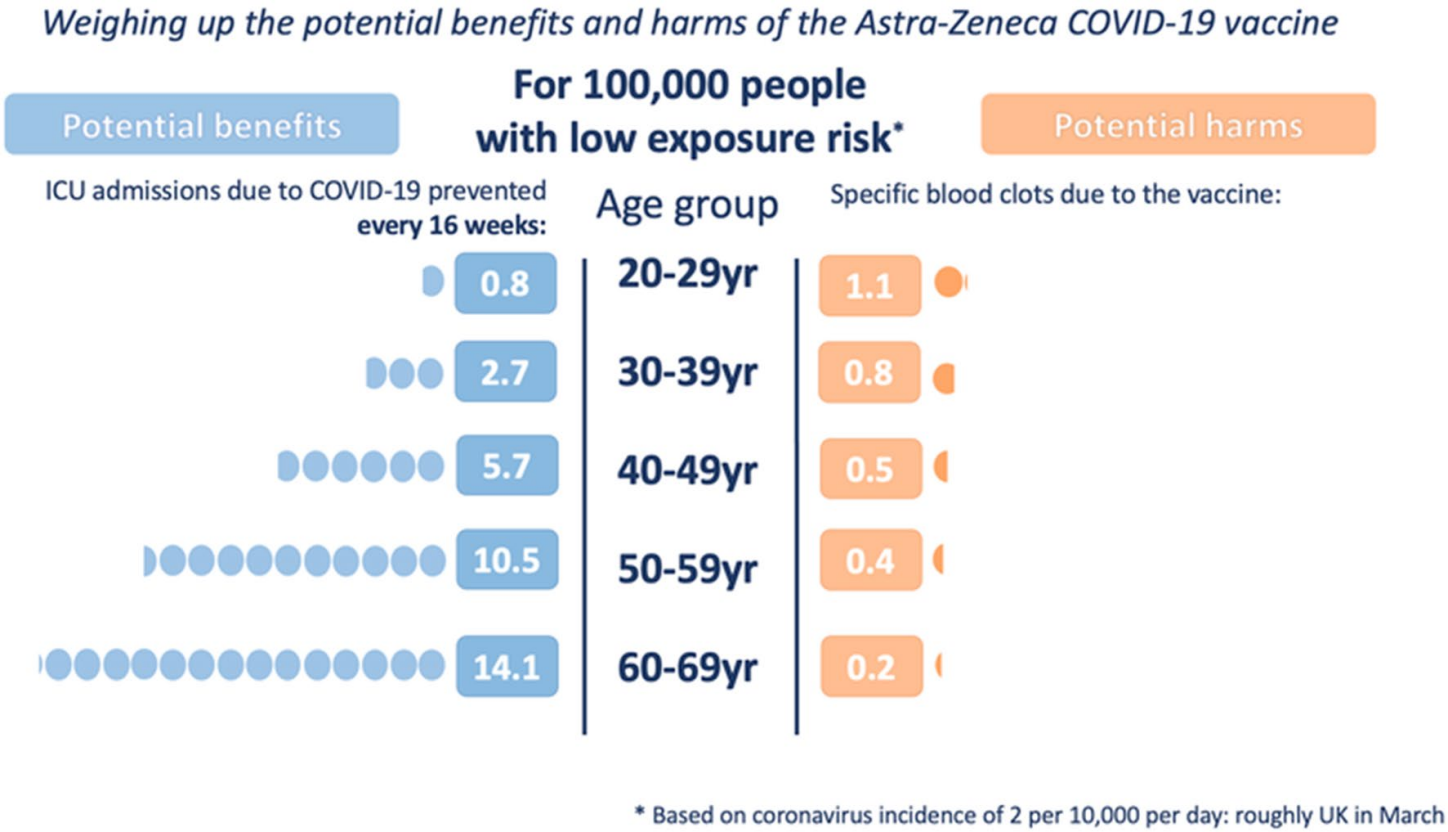
A visual depiction of balancing the benefits and harms when making population vaccination decisions. This shows how recommendations vary by age and prevailing infection rates. Reproduced with permission from A. Freeman and D. Spiegelhalter; for more information, see https://wintoncentre.maths.cam.ac.uk/ accessed 17/06/21

Vaccination policy needs to balance the risks of using versus not using a vaccine, which includes: (i) the risk of otherwise contracting the infection, (ii) the risk of serious complications post-infection, and (iii) whether there is an alternative vaccine with a more favourable side effect profile. This decision must incorporate the prevailing rate of infections, personal comorbidities, and whether any potential vaccine side effects differentially affect certain high-risk groups. This complicates any simple public health decision or message. Communicating this complexity can be aided by visual representations of key factors [such as that used by the UK government communications in Spring 2021 to explain the benefits and harms of the AZ vaccine by age group (see Fig. 3)].

Those who are critical or anti-vaccination will inevitably highlight the potential harms and down-play the benefits. This is a particular issue in the age of enhanced technological and social media accessibility. The general population is faced with a deluge of information rapidly disseminated over the Internet and media. Whilst this ‘infodemic’ may have distinct advantages in logistical organisation and effective public communication, it may also be countered by disinformation spreading “vaccine hesitancy” and threatening uptake [91, 92]. Public education and actively countering misinformation will be essential in increasing worldwide acceptance and vaccine uptake if we are to achieve “herd immunity”.

## Conclusion

Any new vaccine will have been carefully studied in large randomised trials before licencing. These can never be big enough to detect all possible adverse events and national surveillance systems are required to obtain post-marketing data and need to be combined using harmonised datasets that span international boundaries. Such analyses require appropriate governance data sharing and the necessary infra-structure to undertake anonymised individual patient data analyses. However, all post-marketing reports tend to be affected by bias and should be treated with caution with further well-designed and robust studies required to confirm or refute associations between vaccination and adverse events. Importantly, increased rates of adverse events may be due to the disease itself or a higher risk in the population targeted for initial vaccination, even without vaccination. Public messaging must be carefully measured, since the consequences of misreporting may be profound. Even where association is confirmed, contextualisation of risk is important, as risks of infection may far exceed those of adverse events, particularly in periods of high disease transmission. In addition, risks can be effectively mitigated by targeting strategies, timely dissemination of appropriate information, and effective public engagement.

## Data Availability

Not applicable.

## Footnotes

1 Collider bias occurs if an exposure and an outcome both separately cause a third variable (the collider). In this situation, controlling for the collider (whether by study design such as preferential selection of participants with a certain value of the collider, or statistical analysis of participants not lost to follow-up) can generate a biased association between the exposure and outcome when none in fact exists.
